# Going beyond Radial Hydration Models: The Hidden Structures of Chloride and Iodide Aqua Ions Revealed by the Use of Lone Pairs

**DOI:** 10.1021/acs.jpcb.3c06185

**Published:** 2023-12-08

**Authors:** Valentina Migliorati, Paola D’Angelo, Francesco Sessa

**Affiliations:** † Dipartimento di Chimica, “La Sapienza” Università di Roma, P.le Aldo Moro 5, Rome 00185, Italy; ‡ Department of Chemical Sciences, University of Naples Federico II, Comp. Univ. Monte Sant’Angelo, Naples 80126, Italy

## Abstract

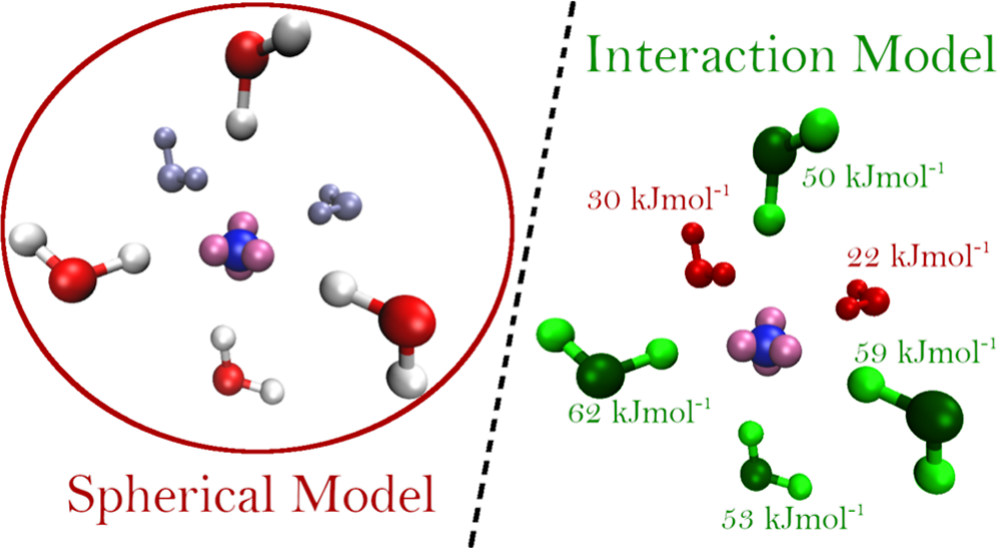

A novel model of hydration for the chloride and iodide ions in water is proposed, which overcomes the limitations of conventional radial models. A new approach, based on a representation of the halide lone pairs, highlighted a subset of first shell water molecules featuring preferential strong interactions with the ion lone pairs, giving rise to tetrahedral hydration structures in both Cl^–^ and I^–^ aqueous solutions. By adopting a novel descriptor correlated to the halide–water interaction energy, we were able to split the conventional first solvation shell into a tight first hydration shell, composed of water molecules strongly interacting with the ions via hydrogen bonds, and a loose first shell containing molecules that are only slightly perturbed by the halide electrostatic charge. The picture emerging from our findings indicates that lone pairs play an important role in the description of systems where hydrogen bonds are the main interactions taking place in the solvation process.

## 1 Introduction

Aqueous solutions of halide ions are of great interest in many physical and chemical processes. Therefore, many investigations have been dedicated to study aqueous solutions of halide ions and to provide a molecular level understanding of halide–water interactions.^1^ A plethora of different techniques have been adopted to characterize the hydration properties of halides, including classical molecular dynamics (MD)^2−10^ and Monte Carlo simulations,^11^ QM/MM simulations,^12−16^ ab initio simulations,^2,14,17,18^ Raman and IR spectroscopies,^19^ X-ray absorption spectroscopy,^10,16,20,21^ and neutron and X-ray diffraction.^22^ Nevertheless, the picture of halide solvation shell structure emerging from such works is inhomogeneous, and first shell coordination numbers and distances reported in the literature are very scattered.^1^ For example, halide–oxygen first shell distance and coordination number are, respectively, in the range 2.70–3.30 Å and 4.0–8.9 for chloride and 3.02–3.70 Å and 4.2–10.3 for iodide.^1,2,8^ There is no consensus in the literature also about the solvation geometry of halide aqua ions: in some investigations, no well-defined structures have been found,^13,16^ while in other studies defined (and flexible) hydration geometries have been reported.^17^ Such variety of results is due to the diffuse character of halide solvation shells that makes it difficult to define them, and also to the fact that the first shell water residence time is very short (picosecond time scale).^1,23^

The solvation of ions is usually described by means of the Wen^24^ and Gurney^25^ model, in which the solvent molecules around the ion are separated into concentric spheres, and each spherical region is composed of molecules assumed to interact in an equal way with the ion. The Wen and Gurney model has provided a very good description of the solvation properties of many monatomic ions in aqueous and nonaqueous solution.^26−33^ However, we have recently proposed an alternative model for the Br^–^ ion in aqueous solution, where the separation between solvent molecules that strongly or weakly interact with the ion is not so neat.^34^ We have indeed shown that bromide hydration can be better described if an approach going beyond the calculation of the solute–solvent distances is used.^34^

Here, we have decided to use this powerful interaction-based approach to study the solvation properties of the Cl^–^ and I^–^ ions in an aqueous solution. Our aim is to understand how changes in the hydrogen bond strength influence the halide hydration process. Therefore, we have chosen two halide ions featuring interactions with water that are both stronger (Cl^–^) and weaker (I^–^) than Br^–^. Our interaction-based approach requires the use of a proper representation of the halide lone pairs and, in the framework of ab initio approaches, such representation can be obtained through the computation of maximally localized Wannier functions centers.^35,36^

In this work, we have thus performed ab initio MD (AIMD)^37^ simulations of the Cl^–^ and I^–^ ions in aqueous solution and using the ion lone pairs in the investigation we managed to identify rather elusive hydration structures. Due to being masked by the inherent disorder of these systems, such structures have not been observed in the literature so far.

## 2 Methods

AIMD simulations of the chloride and iodide ions in water have been carried out using the Car–Parrinello MD (CPMD) method^38^ by adopting the Kohn–Sham density functional theory approach.^39^ The simulations were performed by means of the CPMD package.^40^ The systems were composed of one halide ion and 90 water molecules in a periodic cubic box with a 14 Å edge. As exchange–correlation functional it has been used the BLYP functional.^41,42^ Moreover, dispersion-corrected atom-centered pseudopotentials (DCACP^43^) were adopted for the core electrons of oxygen and hydrogen atoms to provide a correction for the fact that BLYP tends to poorly estimate van der Waals interactions and thus to provide an overstructured liquid water.^44^ By combining BLYP and DCACP pseudopotentials it is possible to overcome such problem, obtaining an improved description of liquid water, as shown in ref (45). Chloride and iodide core electrons are treated instead with Troullier–Martins pseudopotentials.^46^ For the plane-wave basis set, a 70 Ry energy cutoff was used and a fictitious mass of 400 au was adopted to treat the electronic degrees of freedom. The systems were equilibrated in the NVT ensemble (300 K) for 2 ps by means of the Nosé–Hoover thermostat with a coupling frequency of 1500 cm^–1^. A 2 au time step was used, and the production trajectory in the NVE ensemble was carried out for 10 ps. The halide negative charge has been compensated through a homogeneous background charge.^47^ No drift of the fictitious electronic kinetic energy was observed in the course of the simulations. The maximally localized Wannier functions and their centers^35,36^ have been calculated at each time step to obtain a representation of the halide lone pairs. Spatial distribution functions have been computed by means of the TRAVIS code^48^ while all the other analyses have been performed using in-house developed codes. DFT calculations on 2000 isolated ion–water pairs randomly extracted from the CPMD simulations were performed to obtain the ion–water pair interaction energies. The DFT computations were carried out using the same protocol as that adopted in the AIMD simulations.

## 3 Results and Discussion

**Figure 1:**
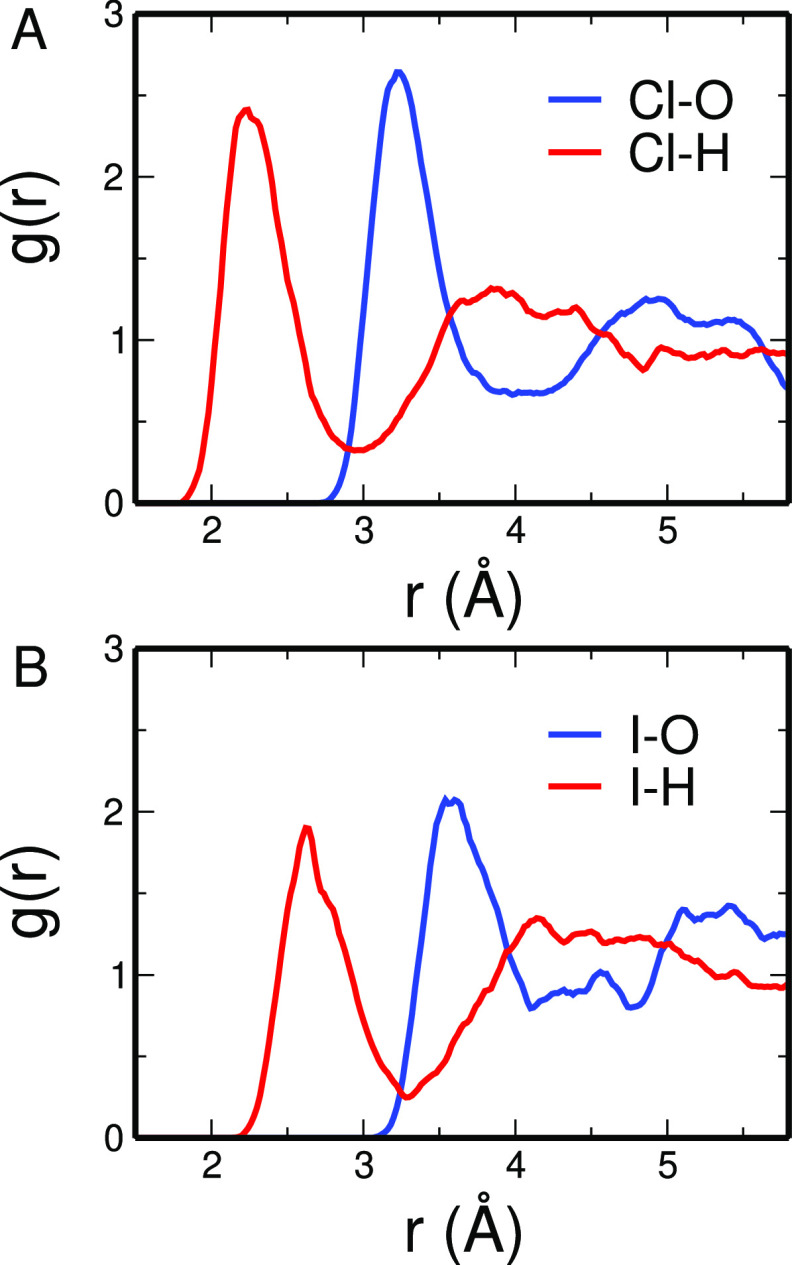
X–O (blue line) and X–H (red line) radial distribution functions calculated from the CPMD simulation of the Cl^–^ (A) and I^–^ (D) aqueous solution.

CPMD simulations of Cl^–^ and I^–^ in water have been carried out using the computational protocol described in the Methods Section and the water structure around halide ions X^–^ has been described, in a first step, by calculating from the MD trajectories the X–O and X–H radial distribution functions (*g*(*r*)’s), which are displayed in Figure 1. In both cases, the X–H *g*(*r*)’s are characterized by two peaks, and the X–O *g*(*r*)’s present a first shell peak comprised between the two peaks of the X–H ones. This result is in line with the fact that usually only one hydrogen atom of the fist shell solvent molecules is pointed toward the halide ion. As concerns the shape of the *g*(*r*), the presence of nonzero minima points to the fact that the first solvation shell is not well defined and water molecules fastly move inside and outside the shell itself. The X–H *g*(*r*) first peaks become less sharp with increasing the halide atomic weight due to the increase in the orientational flexibility of solvent molecules in the first coordination sphere. Moreover, as expected, longer first shell distances are found for I^–^ as compared to Cl^–^, as also evidenced by the X–O *g*(*r*) first peak maximum positions reported in Table 1. Mean hydration numbers obtained by integration of the X–O *g*(*r*) first peak increase with increasing halide atomic weight (see Table 1). The cutoff distances used to calculate the first shell mean hydration numbers are 4.00 and 4.37 Å for Cl^–^ and I^–^, respectively.

**Table 1:** Comparison of the Structural Parameters for the Halide First Solvation Shell Obtained in This Work with the Ranges Reported in the Literature^1,2,8^^a^

halide	*R* (Å)	*R*^lit^ (Å)	*N*	*N*^lit^
Cl^–^	3.21	2.70–3.30	7.2	4.0–8.9
I^–^	3.58	3.02–3.70	8.6	4.2–10.3

a *R* is the halide–oxygen first shell distance, while *N* is the average first shell coordination number.

To compare our findings with the results of previous studies, we list in Table 1 the X–O first shell distance and average coordination number ranges reported in the literature.^1,2,8^ As can be seen, the X–O distances and mean coordination numbers obtained from our calculations fall well within the literature experimental range for both halide ions.

**Figure 2:**
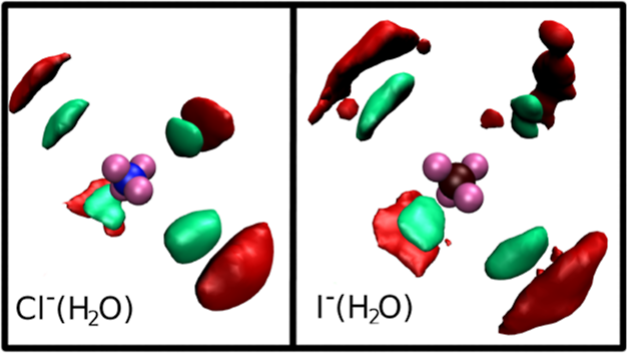
SDFs of oxygen (red surfaces) and hydrogen (green surfaces) atoms around the Cl^–^ (left panel) and I^–^ (right panel) ions. The Cl^–^ and I^–^ ions are shown in blue and bordeaux respectively, while the ion lone pairs are reported in mauve.

As mentioned above, using an approach based on the Br^–^ ion lone pairs, we have recently shown that a subset of first shell water molecules has preferential strong interactions with the Br^–^ lone pairs, forming a short-lived tetrahedral complex around the ion.^34^ To unveil if this peculiar behavior is also shown by Cl^–^ and I^–^ in aqueous solution, we have calculated the spatial distribution functions (SDFs) of oxygen and hydrogen atoms around the Cl^–^ and I^–^ ions, that are shown in panel A and B of Figure 2, respectively. The internal reference system used to calculate the SDFs is constructed using the instantaneous positions of the halide lone pairs. In both cases, four high-probability spots are obtained tetrahedrally arranged along the directions of the halide lone pairs.

Therefore, by use of a reference system based on the instantaneous positions of the halide lone pairs, a strong correlation between the positions of the ion lone pairs and a set of water molecules is obtained. This set of water molecules interacts strongly with the anion lone pairs, generating an instantaneous tetrahedral structure around Cl^–^ and I^–^. By comparing the SDFs obtained for the two halide ions, we can see that the distributions obtained for I^–^ are more disordered than those of Cl^–^. The Cl^–^ SDFs show instead very localized high probability spots as a consequence of stronger ion–water interactions.

Our results thus show that in Cl^–^ and I^–^ aqueous solutions, the disorder of the halide first solvation shell hides a tetrahedral solvation structure. To properly describe such peculiar coordination a simple radial cutoff is not sufficient and a more complex descriptor is needed. We have recently developed a new geometric descriptor, η, which provides a very good description of the hydration properties of Br^–^ in water.^34^ Such descriptor is based on the mutual arrangement of the ion lone pairs and the water hydrogen atoms and it depends on three geometric quantities involved in the hydrogen bonding interaction
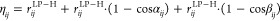
where *i* and *j* label the halide ion and the water molecule interacting with the ion. *r*^LP-H^ is the lone pair-hydrogen distance, α takes account of the orientation of the ion lone pair toward the water hydrogen atom and β instead of the orientation of the water O–H vector toward the ion lone pair (see Figure 2 of ref (34)). The definition of η relies on the fact that hydrogen bonding is the most significant interaction taking place in the hydration process of halide ions. In general, two species forming a hydrogen bond interact with each other through a hydrogen atom and the electron density of the acceptor atom, which can be represented by its lone pairs. In particular, η can be considered as a weighted distance between the halide lone pair and the hydrogen atom of the water molecules which interacts with such a lone pair. The definition includes two angular weights taking into account the mutual orientations of the lone pair and of the O–H vector in such a way that the more linear the configuration, the lower the values of the angular weights. Stronger interactions are therefore represented by lower η values.

**Figure 3:**
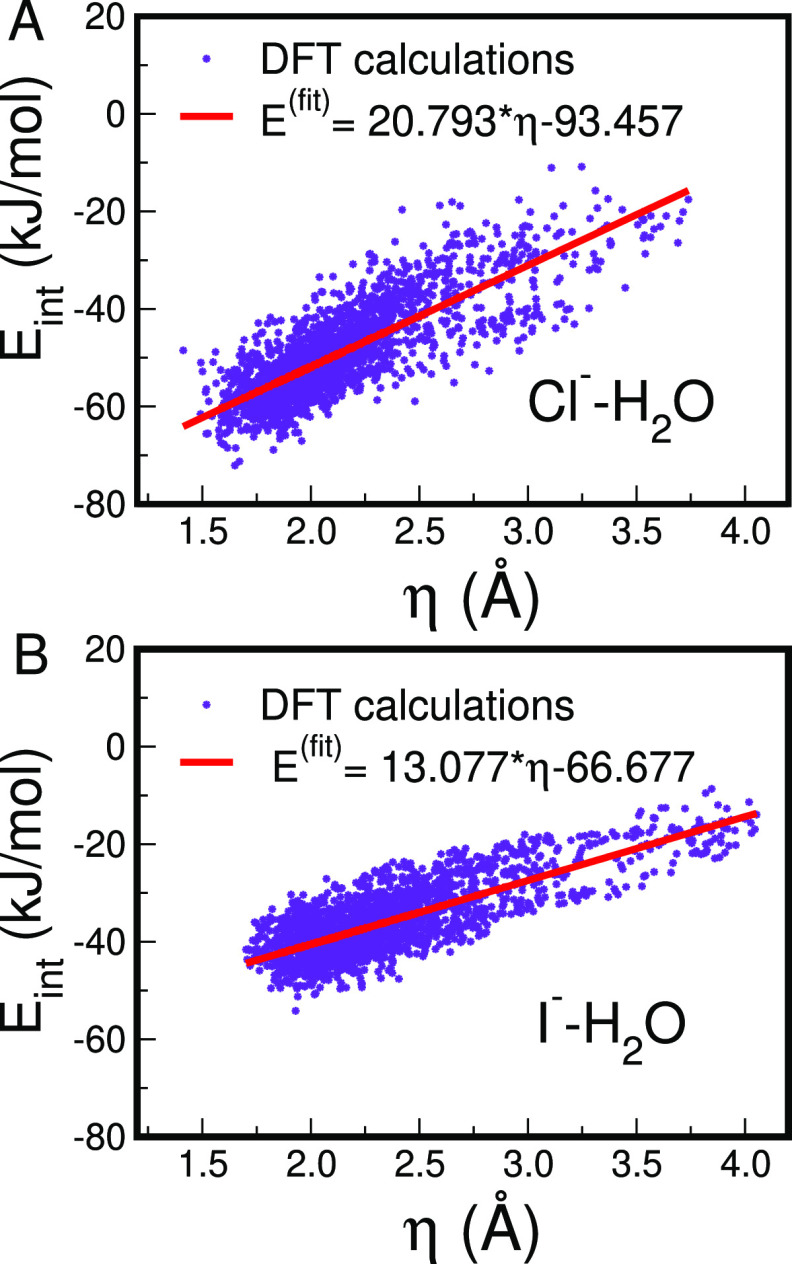
Interaction energies against η values calculated for 2000 ion–water pairs randomly extracted from the CPMD simulation of the Cl^–^ (A) and I^–^ (B) aqueous solution. The red lines are the linear regressions of the energy data sets, whose fitting equations are shown in the legends.

η should thus correlate with the pair interaction energy between the halide ion and the water molecule. To demonstrate that this is the case, 2000 Cl^–^ or I^–^–water pairs have been extracted, and their interaction energies have been computed through DFT calculations. The η value of the ion–water pair has been then recalculated and the interaction energies as a function of η have been plotted (see Figure 3A,B for Cl^–^ and I^–^, respectively). The correlation between η and the pair interaction energies can be clearly observed for both the Cl^–^ and I^–^ ions. The linear regressions of the two data sets are also shown in the figure and the correlation obtained can be appreciated by evaluating the correlation coefficient (*R*^2^) between data and the linear regression which is 0.81 and 0.79 for Cl^–^ and I^–^, respectively. Note that we have reported the fitting equations in the figure legends and that such equations can be very useful to convert η values to estimated energies. The root mean square errors between the data and the regression model are instead 5.6-kJ/mol for the Cl^–^ ion and 4.4 kJ/mol in the I^–^ case, which is an optimal target for a descriptor designed to estimate the strength of an interaction. Indeed, such values are similar to the value of 5 kJ/mol, which has been estimated as the “optimistic” error which is obtained when calculating bond dissociation energies by means of electronic structures methods.^49^

**Figure 4:**
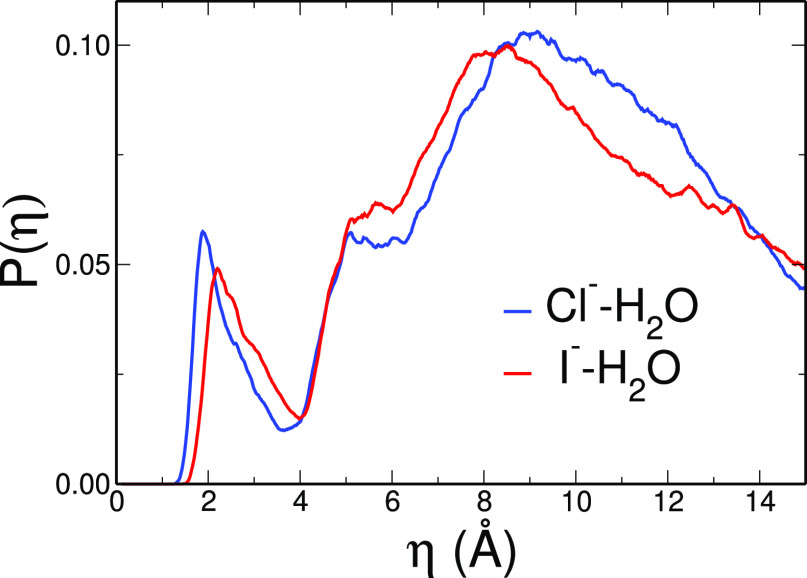
η distribution *P*(η) calculated for all ion–water pairs in the CPMD simulation of the Cl^–^ (blue curve) and I^–^ (red curve) aqueous solution.

After checking the capability of the η descriptor to correlate with the ion–water interaction energies of isolated pairs, we evaluated its ability in the aqueous solution by computing the η distribution function *P*(η) of all ion–water pairs in the system throughout the AIMD trajectories (see Figure 4). The obtained functions show striking similarities with halide–water pair energy distributions calculated by Chandrasekhar et al. starting from classical MD simulations.^50^ Indeed, in both Chandrasekhar’s and η distributions a small first peak at low energies/low η is present, which is due to water molecules belonging to the ion first solvation shell, and a wide high energy peak arising from all of the other solvent molecules.

η values can be converted to energies through the regression equations reported before. In terms of energy, the first peak maxima of the η distributions are located at 54(±6) and 38(±4) kJ/mol for Cl^–^ and I^–^, respectively (corresponding to η values of 1.88 and 2.20 Å). These values are in very good agreement with the experimental values of halide–water binding energies reported by Hiraoka et al.^51^ In their work, Hiraoka et al. provide the enthalpies of formation of halide hydration complexes as a function of the hydration number.^51^ Here, as an experimental estimate of the ion–water interaction energy, we averaged the enthalpy changes for the stepwise addition of the first four water molecules to the ion. The resulting ion–water interaction energies are 53(±2) and 40(±1) kJ/mol for the Cl^–^ and I^–^ ions, respectively.^51^ By comparing the η results obtained for the two halides, we can see that the η distribution related to the iodide first solvation shell is shifted toward larger η values as compared to chloride. This is in line with the existence of weaker halide–water interactions, as expected following the increase in halide atomic weight.

**Figure 5:**
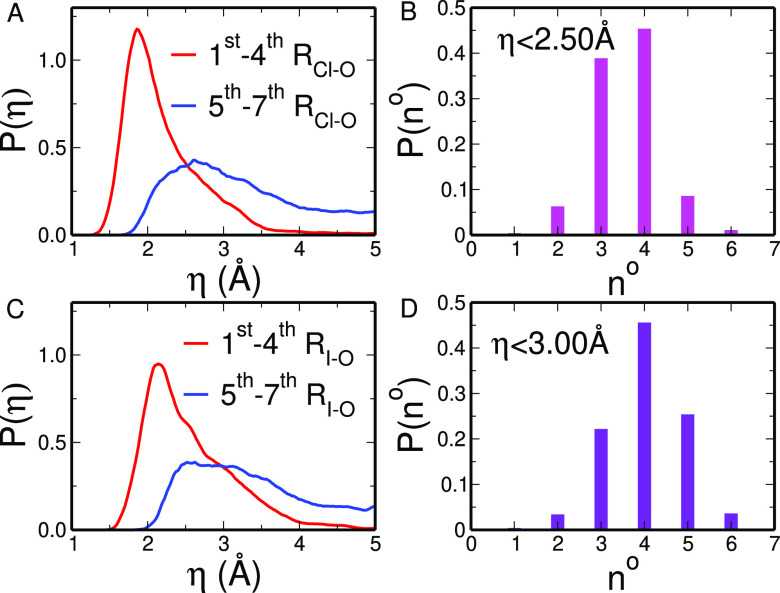
(A/C) η distributions for two selections of water molecules: 1st to 4th closest to the ion (red line), and 5th, 6th and 7th closest to the ion (blue line). The distributions were calculated from the CPMD simulations of the Cl^–^ (A) and I^–^ (C) aqueous solution. (B/D) Instantaneous coordination number distributions for the Cl^–^ (B) and I^–^ (D) ions evaluated by selecting all the water molecules having an η value lower than 2.50 and 3.00 Å for Cl^–^ and I^–^, respectively.

Figure 5A,C shows the η distributions (calculated for Cl^–^ and I^–^, respectively) for the four nearest molecules to the halide ion and the 5th to 7th molecules in terms of X–O distance. A sharp peak (with maxima at 1.86 and 2.14 Å for Cl^–^ and I^–^, respectively) is found for the four molecules closest to the halide. Note that again the I^–^ distribution is shifted to larger η values, as iodide forms weaker interactions with water as compared to chloride. The distributions drop to zero at about 4.0 and 5.0 Å for the Cl^–^ and I^–^ ions, respectively. The 5th–7th nearest molecules give instead rise to a very broad peak with low intensity which is shifted toward larger η values. Such findings show that the external solvent molecules of the first coordination sphere form much weaker interactions with the ions. However, for both halides the 1st–4th and 5th–7th distributions significantly overlap suggesting that the 5th–7th water molecules can occasionally interact with the halide ion more strongly than the closer solvent molecules.

By considering all together these results, we propose a model for the Cl^–^ and I^–^ solvation structure in which the standard first solvation shell defined by a simple cutoff on the ion–water distances can be split into a “tight” first shell of molecules forming strong interactions with the halide and a “loose” first shell, containing the remaining first shell water molecules less interacting with the ion. To provide a quantitative separation of the two subshells, we have used a cutoff on the η value rather than on the distance. We chose η cutoff values of 2.50 and 3.00 Å for Cl^–^ and I^–^, respectively, which correspond to the η value at which the 1st–4th and 5th–7th distributions intersect with each other. In this way, close molecules interacting poorly with the ion will not be included in the tight first shell. Instead, farther molecules that show strong interactions with the ion will be part of the tight first solvation shell. By calculating the halide–water coordination number distributions with the chosen η cutoff (see Figure 5B,D for Cl^–^ and I^–^, respectively), a dominant 4-fold cluster is obtained for the tight first solvation shell of both halide ions, in line with the existence of tetrahedral hydration geometries.

**Figure 6:**
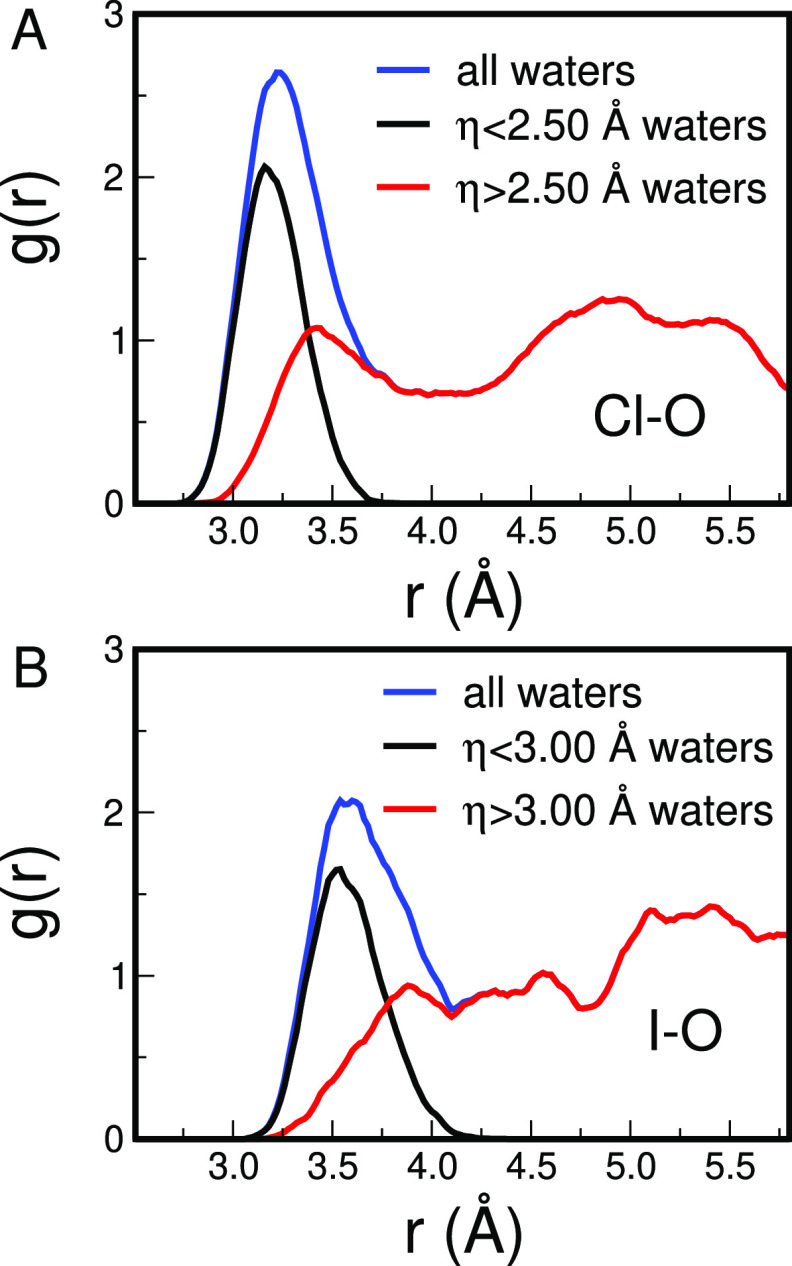
X–O radial distribution functions *g*(*r*)’s evaluated for water molecules below (tigh shell, black line) and above (loose shell + bulk, red line) the chosen η cutoff value calculated from the CPMD simulations of the Cl^–^ (A) and I^–^ (B) aqueous solution. The total X–O *g*(*r*)’s are also reported as blue curves.

It is interesting to calculate the separate contributions to the X–O *g*(*r*) of the solvent molecules belonging to the tight first shell and of the other molecules, including the loose solvation shell and the bulk water. The results of this analysis are shown in Figure 6A,B for the Cl^–^ and I^–^ ions, respectively. For the tight solvation shell, sharp peaks are found with maxima located at 3.16 and 3.54 Å for the Cl^–^ and I^–^ ions, respectively. Note that the most probable distances of tight shell water molecules are shorter than those of the conventional solvation shell listed in Table 1, as they form stronger interactions with the halide ion. The structuredness of the obtained peaks is further proof of the existence of a hidden structure formed by the solvent molecules belonging to the tight coordination shell. Interestingly, the *g*(*r*)’s smoothly go to zero at about 3.75 and 4.25 Å for Cl^–^ and I^–^, respectively; indeed, the functions show no truncation, even if solvent molecules at longer distances (up to about 4.0 and 4.5 Å for Cl^–^ and I^–^, respectively) could have been included in the chosen η cutoff value. As far as the loose shell is concerned, a much disordered and unstructured function is obtained, which is shifted toward larger distances as compared to that of the tight solvation shell. The overall shapes of the loose shell + bulk *g*(*r*)’s point to the fact that bulk solvent molecules go into the first shell space region but are not perturbed in a significant way by the halide. Note that such molecules occasionally move close to the ion even if they form weak interactions with it. This is further evidence that the halide solvation process in water is not well described using conventional models based only on distance criteria.

**Figure 7:**
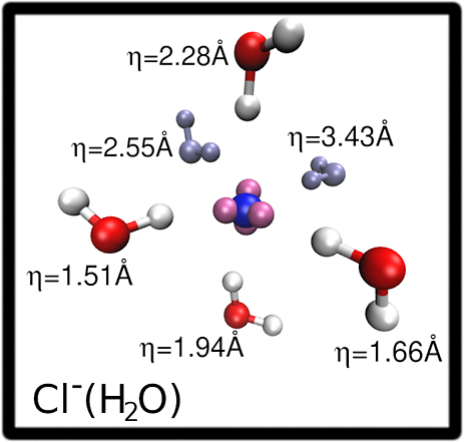
Simulation snapshot extracted from the trajectory of the Cl^–^ ion in aqueous solution displaying how the conventional first hydration shell is divided into a tetrahedral tight hydration shell formed by the water molecules strongly interacting with the chloride lone pairs (the oxygen and hydrogen atoms of such water molecules are shown in red and white, respectively) and a loose hydration shell of undefined geometry (water molecules colored iceblue). The Cl^–^ ion is shown in blue, while its lone pairs in mauve. The η values of the water molecules are also reported.

To give an idea of the separation between tight and loose solvation shells, we have extracted one simulation snapshot of the Cl^–^ first shell cluster as an example. The cluster is shown in Figure 7 and includes all of the water molecules belonging to the Cl^–^ first solvation shell as conventionally defined inside the cutoff distance. It is possible to observe from the figure how the conventional first hydration shell is divided into a tetrahedral tight hydration shell formed by four water molecules strongly interacting with the chloride lone pairs and a loose hydration shell of undefined geometry that does not show preferential interactions with the ion lone pairs. The η values of the Cl^–^ first shell molecules are also reported in the figure, showing that a cutoff on the η descriptor is ideally suited to distinguish between the tight and the loose shell molecules. Indeed, all the tight shell molecules show low η values while the loose ones have higher values outside the chosen η cutoff.

**Figure 8:**
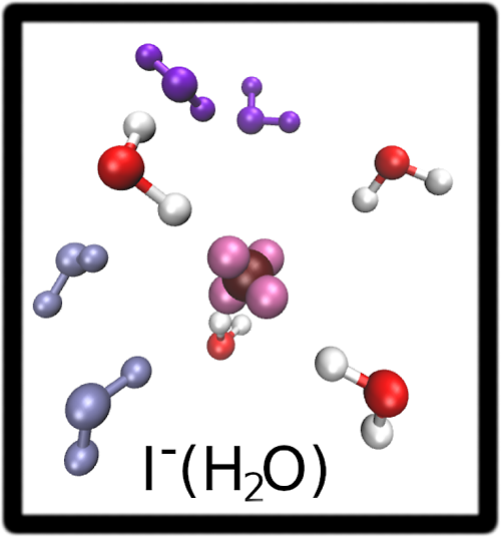
Simulation snapshot extracted from the trajectory of the I^–^ ion in aqueous solution displaying how the conventional first hydration shell is divided into a tetrahedral tight hydration shell formed by the water molecules strongly interacting with the iodide lone pairs (the oxygen and hydrogen atoms of such water molecules are shown in red and white, respectively) and a loose hydration shell of undefined geometry (water molecules colored iceblue). Two additional water molecules colored purple do not interact with iodide, pointing their hydrogen atoms away from the I^–^ ion.

In the case of iodide, the picture is even more complex. Within the conventional first shell cutoff distance, we found, among loose shell molecules, molecules that should be considered “second shell molecules” in a more general sense. Indeed if we look at the simulation snapshot of an I^–^ first shell cluster shown in Figure 8, we clearly observe a tetrahedral tight hydration shell formed by four water molecules strongly interacting with the iodide lone pairs and a loose hydration shell composed of molecules weakly interacting with the I^–^ lone pairs. However, two additional water molecules are present that cannot be structured by the ion. Indeed, by pointing their hydrogen atoms away from I^–^, such molecules do not show the proper orientation to be solvating the anion. These water molecules are “fortuitously” located inside the I^–^ cutoff distance which defines the I^–^ conventional first solvation shell. Rather than interact directly with the anion, these water molecules are bound to the ion tight first shell instead. In this sense, they are better described as second-shell water molecules. All together, these results show how the complexity of halide hydration cannot be accounted for by conventional models which distinguish among different solvation shells by considering only halide–water distances.

Lastly, we analyzed the exchange dynamics of the solvent molecules composing both tight and loose hydration shells of the halide ions with the bulk of the solvent. We sampled separately different kinds of solvent exchange events, namely between: tight and loose shells, loose shell and bulk, and tight shell and bulk. In particular, the exchange events between tight shell and bulk have been monitored to understand whether they happen directly or via the loose shell. Note that by bulk here we refer to any water molecule beyond the first minimum of the halide-oxygen radial distribution function (4.00 and 4.37 Å for Cl^–^ and I^–^, respectively). In monitoring exchange events, we employed a threshold time of 0.1 ps to screen for temporary fluctuations: if an exchanging water molecule returns to its former region within this threshold time, then the event is discarded as a temporary fluctuation. For both ions, Table 2 shows that a larger amount of exchange events happen between the loose shell and bulk molecules. A lower, although still significant, amount of exchanges is also found between tight and loose shell molecules. Interestingly, the amount of exchanges between tight shell and bulk water molecules is much lower, and all of those exchanges happen via the loose shell region. In other words, tight shell molecules are required to free themselves from the tetrahedral complex to exchange with the bulk. The different exchange dynamics of tight and loose hydration shells is also highlighted by the different normalized mean residence time (NMRT), which we computed using the “direct method” proposed by Hofer et al.^52^
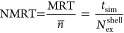
1where MRT is the water mean residence time in a given shell, *n̅* is the average coordination number of the shell, and *N*_ex_^shell^ is the total number of exchange events involving the shell. The evaluated NMRTs are shown in Table 2 for the hydration shells of both ions. The results suggest a different dynamic behavior between tight and loose shell water molecules. However, note that while tight shell water molecules have a reduced mobility as compared to the loose ones, the interconversion between the tight and loose hydration shells is still fast enough to result in a very disordered conventional first shell.

**Table 2:** Number of Exchange Events, *N*_ex_ between: Tight Shell and Loose Shell (T–L), Loose Shell and Bulk (L–B), Tight Shell and Bulk Directly (T–B), and Tight Shell and Bulk Through the Loose Shell (T–L–B)^a^

	*N*_ex_				NMRT	
	T–L	L–B	T–B	T–L–B	T	L
Cl^–^	48	98	0	11	0.17	0.06
I^–^	32	108	0	8	0.25	0.07

a Normalized mean residence time, NMRT (ps), are also reported for tight (T) and loose (L) shells.

## 4 Overview and Conclusions

In this work, we have performed an accurate characterization of the Cl^–^ and I^–^ solvation properties in water by adopting AIMD and novel approaches based on the use of the halide lone pairs. The first important finding we have obtained is that chloride and iodide in water form tetrahedral hydration structures. More specifically, they form a tetrahedral tight first shell which includes molecules having strong hydrogen bond interactions with the halide. The other solvent molecules that are located in the first shell distance region, forming the so-called loose hydration shell, are unstructured solvent molecules only slightly perturbed by the halide electrostatic charge. These results are in line with those previously obtained by us for the Br^–^ ion in aqueous solution.^34^ Moreover, in the case of iodide, which is the largest (and so most diffuse) halide ion forming the weakest interactions with water molecules, the separation goes beyond the tight and loose hydration shells. The conventional first solvation shell (defined by a distance cutoff) also contains water molecules that do not have the proper orientation to interact with the iodide ion, pointing their hydrogen atoms away from I^–^. Instead, these water molecules interact with other water molecules bound to the ion. This finding is further evidence of how the complexity of halide hydration cannot be accounted for by conventional models which distinguish among different solvation shells by considering only halide–water distances.

One important remark we would like to make concerns the halide electron density. In general, any isolated atom or ion is spherical. This notion can be tricky to reconcile with the chemical concept of nonspherical atomic orbitals. A way to explain this is to borrow another fundamental chemical concept: hybridization. Let us use a simple example, an excited hydrogen atom in vacuum. The single electron occupies a 2p orbital, but due to degeneracy we describe the electron as a “resonance hybrid” of 2p_*x*_, 2p_*y*_, and 2p_*z*_. Such a description results in the atom’s electron density having spherical symmetry. A multielectron case, while more complicated, bears the same conclusion: quantum mechanics does not yield a directional atom in a nondirectional space. On the contrary, under an anisotropic influence (e.g., the electric field generated by another atom), what happens depends on the symmetry of occupied orbitals. In other words, if an anisotropic environment removes the orbital degeneracy, then the atom is no longer necessarily spherical. This is the case for the halide ions in water. Due to the presence of the surrounding water molecules, the degeneracy is broken along with the spherical symmetry of the anion electron density. As a consequence, when the halide ion interacts with the water molecules, the tetrahedral symmetry of the halide lone pairs induces a tetrahedral arrangement of the water hydrogen atoms, as we have shown in this paper.

The peculiar hydration structures that emerged from this work have never been found in the literature so far since both theoretical and experimental techniques have always described ion solvation using radial models. From an experimental point of view, structural experimental techniques such as X-ray or neutron diffraction and extended X-ray absorption fine structure (EXAFS) usually describe the solvation process using *g*(*r*) functions. Furthermore, it is important to stress that each structural experimental technique available has its own limitations when applied to determine the coordination numbers of monatomic ions in solution. The ideal condition to estimate the coordination number of a monatomic ion in solution would be the hypothetical state of infinite dilution. In practice, this can be approximated by studying diluted solutions (≤0.1 M). However, most of the experimental results on ion hydration have been obtained by means of X-ray (or neutron) diffraction techniques, which require highly concentrated samples (1–5 M) due to their low selectivity. These conditions result in very large uncertainties on the obtained coordination numbers, as shown by the large spread of data found in the literature. For example, the range of coordination numbers found using X-ray or neutron diffraction for Cl^–^ in water goes from 4.2 to 8.9.^1^ For I^–^ in aqueous solution, the spread is even slightly larger, going from 4.2 to 9.6.^1^ On the other hand, EXAFS high selectivity makes it the technique of choice to study the solvation properties of monatomic ions, allowing one to investigate also very dilute solutions (down to the millimolar concentration range) and to determine the distances between the ions and the coordinated ligands with very high accuracy. However, when disordered systems are studied, there is a very large correlation between the coordination numbers that can be obtained from the analysis of EXAFS spectra and the Debye–Waller factors. As a consequence, the coordination numbers are still affected by large uncertainties and cannot be unambiguously determined from the analysis of the EXAFS data. Moreover, additional issues arise when treating halide ions in water due to the diffuse character of the solvation shells, where the EXAFS technique is not able to distinguish between strongly and weakly coordinated solvent molecules. Therefore, in the study of halide hydration, the use of theoretical approaches is particularly important. Computational techniques allow one to go beyond the current experimental limitations, providing answers to the elusive hydration structures formed by halide ions in water.

However, also from a theoretical point of view chloride and iodide hydration has always been treated using radial models, implicitly assuming the correctness of the model of Wen^24^ and Gurney.^25^ Conversely, we propose here an alternative model of halide hydration in which the separation between solvent molecules that strongly or weakly interact with the ion cannot be obtained on the basis of just solute–solvent distances, but rather, they can be distinguished by adopting a new analysis procedure which makes use of the halide lone pairs. In particular, we have adopted a novel geometrical descriptor, η, able to describe a linear interaction between two species. Such a descriptor indeed correlates with the ion–water pair energy, and using a cutoff on the η value, it is possible to discern between the solvent molecules forming either strong or weak interactions with the ion. For such characteristics, we suggest that η can be adopted, in general, also to determine if two species are hydrogen bonded, providing an improved description of hydrogen bonding than simple geometric criteria. Indeed, the presence of a hydrogen bond is usually established by adopting sharp cutoffs on several geometric parameters and such procedure can be a crude approximation in the description of properties that are instead continuous. In this respect, the application of a cutoff on η, which is a function depending on all of the geometric parameters, represents an improvement of the description, as this would be a continuous extension of multiple cutoffs. Moreover, η has shown a strong correlation with the ion–water pair interaction energy, which is the most relevant quantity in defining an interaction. Note indeed that some hydrogen bond definitions are based on energetic criteria, and the use of a cutoff on the η values can represent, to some respects, the simultaneous application of energetic and geometrical criteria.
